# Role of the mucin-like glycoprotein FCGBP in mucosal immunity and cancer

**DOI:** 10.3389/fimmu.2022.863317

**Published:** 2022-07-22

**Authors:** Qiao Liu, Xia Niu, Yang Li, Jia-rui Zhang, Shao-jun Zhu, Qi-yuan Yang, Wei Zhang, Li Gong

**Affiliations:** ^1^ Department of Pathology, The Second Affiliated Hospital of Air Force Medical University, Xi’an, China; ^2^ College of Animal Science and Technology, Northwest A&F University, Yangling, China; ^3^ Department of Molecular, Cell and Cancer Biology, University of Massachusetts Medical School, Worcester, MA, United States

**Keywords:** mucosal immunity, MUC2, FCGBP, TFF family, SARS-CoV-2, EMT, tumor immunity

## Abstract

IgGFc-binding protein (FCGBP) is a mucin first detected in the intestinal epithelium. It plays an important role in innate mucosal epithelial defense, tumor metastasis, and tumor immunity. FCGBP forms disulfide-linked heterodimers with mucin-2 and members of the trefoil factor family. These formed complexes inhibit bacterial attachment to mucosal surfaces, affect the motility of pathogens, and support their clearance. Altered FCGBP expression levels may be important in the pathologic processes of Crohn’s disease and ulcerative colitis. FCGBP is also involved in regulating the infiltration of immune cells into tumor microenvironments. Thus, the molecule is a valuable marker of tumor prognosis. This review summarizes the functional relevance and role of FCGBP in immune responses and disease development, and highlights the potential role in diagnosis and predicting tumor prognosis.

## Introduction

The purpose of mucosal barriers is to maximize protection against harmful substances entering the digestive and respiratory systems, help to recognize invading cellular components, and respond rapidly under such attacks (1). The mucus secreted by epithelial cells is crucial for the functioning of these barriers (2), which act as a first line of defense against foreign pathogens. Mucus protects the intestine from harmful antigens and pathogens and provides a physiologic barrier against the harsh luminal environment. Mucins are the main components of mucus, with high-molecular-weight mucins being responsible for maintaining the viscoelasticity of the mucus barrier (3). Mucins are widely expressed by epithelial tissues. They are characterized by a variable number of tandem repeat peptide sequences rich in proline, threonine, and serine and show extensive O-linked glycosylation.

To date, more than 20 mucins have been identified. Depending on whether they form sticky extracellular secretions or viscoelastic polymer gels, they are classified as either membrane-bound or secreted mucins (4). Goblet cells are specialized epithelial cells responsible for the production of the airway and intestinal mucus barrier (5). Classic secretory mucins produced by intestinal goblet cells include mucin (MUC)-2, MUC5AC, MUC6, and MUC5B. FCGBP is a mucin secreted by small intestinal and colonic goblet cells, as well as salivary mucus glands (3, 6). FCGBP binds to the FC portion of Immunoglobulin G (IgG), but does not interact with IgA, IgM, or IgG-Fab. It does not cross-react with antibodies against Fcγ receptor I, II, or III found on neutrophils and macrophages (7, 8). FCGBP secreted by goblet cells enter the intestinal lumen together with mucus. It is an important part of mucosal immune defense, and participates in protective immunity and intestinal inflammation (9). The tissue distribution of FCGBP messenger RNA (mRNA) is primarily studied by Northern blotting (8). It is reported that it is expressed in the colonic epithelium, placenta, and amniotic epithelium (10). Immunostaining also demonstrates that FCGBP expression is detected in various tissues that produce mucus, such as the intestinal and colonic epithelium, gallbladder, bladder, bronchi, submandibular glands, and cervix (11). FCGBP is distributed in the whole-body mucosa, and its function is similar to the secreted mucins MUC2, MUC3 and MUC4. In other words, it fulfills a cytoprotective and anti-inflammatory role in tissues.

This review is divided into three parts. It will analyze the structure of FCGBP and its relationship with partner genes, and introduce in detail the role of FCGBP in the functioning of the mucus barrier. Of course, the broad relevance of FCGBP in tumor research is also discussed, to provide new insights into the various functions of this molecule.

## Structural features of IgGFc-binding protein

**Figure 1:**
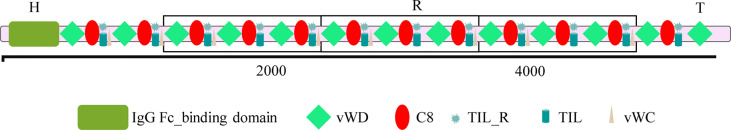
The structure of IgGFc-binding protein (FCGBP). vWDs, von Willebrand factor type D domains; C8, contains 8 conserved cysteine residues domains; TIL, trypsin inhibitor–like cysteine-rich domains; TIL-R, reactive site on conserved domain TIL; vWCs, von Willebrand factor type C domains.

The full-length complementary DNA (cDNA) of FCGBP was first cloned by Harada et al. in 1997 from the human colonic epithelium (8). The gene coding for FCGBP is located on chromosome 19q13. The full-length cDNA is 17,000 bp, of which 16,200 bp represent the coding region (CDS). Human FCGBP contains 5,405 amino acids with a calculated molecular weight of 500 kDa. Using different methods, various forms of FCGBP were detected, with a molecular weight of 200, 100, 70–80, and 55 kDa (12, 13). These discrepancies in molecular weight may be caused by the autocatalytic cleavage of FCGBP, post-transcriptional modifications, or post-translational protease hydrolysis (13). The existence of different forms of FCGBP has been clearly detected in different tissues and organs (14). We analyzed the FCGBP amino acid sequence for the presence of known conserved domain structures using a computer algorithm provided by National Center for Biotechnology Information (NCBI) [NCBI Conserved Domain Search (nih.gov)]. Simultaneously we detected a single IgG Fc-binding domain, 13 von Willebrand factor type D (vWD) domains, 12 trypsin inhibitor–like cysteine-rich (TIL) domains, 12 reactive sites on conserved domain TIL (TIL-R), 12 ‘contains 8 conserved cysteine residues’ (C8) domains, and 8 von Willebrand factor type C domains. The schematic diagram of the structure of FCGBP, based on this analysis, is shown in **Figure 1**.

Structurally, FCGBP may be divided into three main parts the H, R, and T domains. The unique H domain, containing the N-terminal IgG Fc-binding domain, is composed of 450 amino acids. The R domain, contributing to the majority of the FCGBP structure, is composed of 12 tandem repeats, composed of vWD-C8-TIL-TILR domain repeats (15). Finally, the T domain is a C-terminal vWD domain, consisting of 170 amino acids. The unique H domain has a low cysteine content and may have an important role in the formation of the biologically active mature protein. It has been suggested that the H domain may be involved in processing FCGBP peptides into active forms with an Fc-binding activity. Alternatively, it may aid the intracellular targeting of the protein for processing in the Golgi apparatus. The deletion of the H domain results in the loss of the Fc-binding activity. The R domain is rich in cysteine residues, has typical recognition sites for glycosylation, and is sensitive to periodate and hydrogen peroxide treatment, implying that FCGBP is highly glycosylated. In the early stages of biosynthesis in the endoplasmic reticulum, the vWD domain readily undergoes autocatalytic cleavage at the GDPH (Gly-ASP-Pro-His) sequence, forming FCGBP fragments with different molecular weights (16). The C-terminal GDPH cleavage product generates a reactive anhydride, which can react with other molecules to form a covalent ester or amide bond, while the N-terminal sequence, in the absence of a covalent bond, will be released after reduction (17).

The structure of FCGBP is similar to that of mucin-related proteins reported. Together with its intracellular transport and localization in goblet cells, this suggests that FCGBP may be a component of mucus (12). Gelatin-forming mucin is generally considered to be a large molecule formed by linking together vWD domains *via* dozens of cysteine residues (18). For example, the tandem repeat domains of mucins MUC2 (18), MUC3 (19), MUC4 (20), and MUC6 (21) consist of the repeats of 17 amino acid units, a characteristic feature of traditional mucins. Similarly, the R domain of FCGBP also contains short repeat units. FCGBP also exhibits other typical characteristics of mucins, including a high molecular weight (>200 kDa), intramolecular S-S bonds, cysteine-rich domains, high glycosylation, and secretion from goblet cells into the intestine (8, 12). Therefore, FCGBP is classified as mucin-like protein, serving as a gel-like component of mucosa and participating in the maintenance of mucosal structure. Apart from the production of FCGBP (22), goblet cells also secrete proteins such as MUC1/2/3/4/5/5AC/5B/6/7, calcium-activated chloride channel regulator 1 (CLCA1) (23), anterior gradient 2 (AGR2) (24), zymogen granule 16 (zg16) (25), and trefoil factor 3 (TFF-3) (26) to form the gastrointestinal mucus skeleton (27), enhancing the mucus barrier and its antibacterial effect. Bacterial metabolites, such as short-chain fatty acids, mainly butyric acid, propionic acid, and acetic acid, stimulate goblet cells, increasing the expression of MUC2 and TFF genes *via* G protein–coupled receptors (17). FCGBP often interacts with MUC2 and TFFs, forming heterodimers. At the same time, FCGBP has a significant similarity with the von Willebrand factor (vWF). FCGBP, MUC2, and vWF all have a conserved amino acid domain containing the sequence CGLCGN (8). This is a characteristic motif found in thioredoxin (28) and protein disulfide isomerase (29). This thioredoxin motif (CGLC) is present in each vWD domain of FCGBP, although its function or relevance remains to be determined.

## Role of IgGFc-binding protein in mucosal defenses

**Figure 2:**
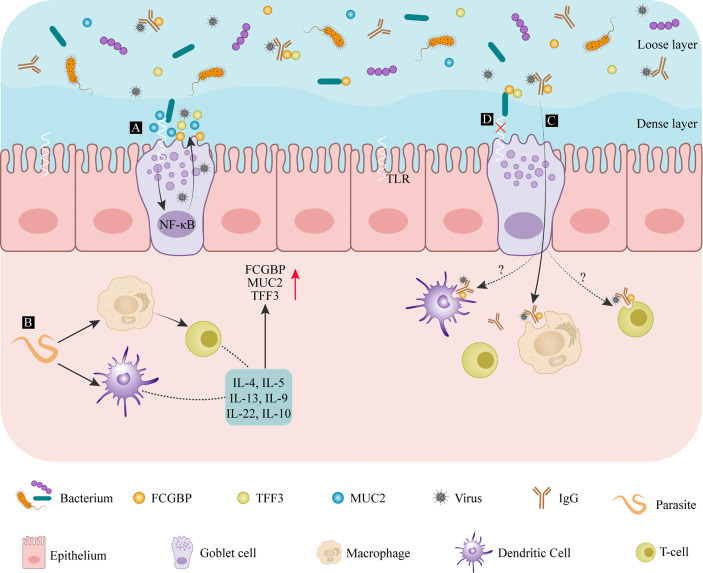
The defense mechanism of FCGBP in mucosal diseases of the colon. **(A)** After entering the intestinal dense mucosa, bacterial compounds bind to Toll-like receptors, activating the NF-κB signaling pathway and promoting further mucus release. **(B)** After microorganisms, such as parasites, are detected by DCs or macrophages, their antigens are presented to T cells where they induce cytokines such as IL-13 and other cytokines secretion, promoting FCGBP production. **(C)** After FCGBP interacts with an IgG antigen complex, it is transported across the mucosa and is recognized by macrophages. **(D)** FCGBP-TFF3 complexes bind to bacteria to prevent them from passing through the epithelial barrier. IL, interleukin; FCGBP, IgGFc-binding protein; MUC2, mucin-2; TFF, trefoil factor family; TLRs, Toll-like receptors.

The density of goblet cells among the intestinal epithelial cells increases gradually from the duodenum, toward the jejunum, intestinal colon, and distal colon (2). The wall of the small intestine is covered in a loose mucus layer, while the large intestine contains a loose and a dense layer of mucus (27) as illustrated by different shades of blue in **Figure 2**. Microorganisms in the intestinal lumen, shown in orange, purple, and blue in **Figure 2**, are mainly present in the loose mucus layer. The density of intestinal colonization with bacteria shows the same increasing gradient seen with goblet cells (2). When pathogenic microorganisms, such as parasites, bacteria, or viruses, are in contact with the intestinal epithelium in large quantities, this will trigger a pronounced immune response by the host, resulting in the production of large amounts of mucus. FCGBP is one of the early response genes after microbial infection (30), and its expression is strongly induced by cytokines.

Toll-like receptors (TLRs) are transmembrane receptors located on the cell surface or in intracellular endosomes. These receptors are activated by bacterial lipopolysaccharide (LPS) components, as *Citrobacter rodentium* (31). Their presence facilitates goblet cell differentiation and mucus secretion by triggering the nuclear factor-k-gene binding (NF-κB) pathway (32) (**Figure 2A**). The immune response against intestinal worms as *Nippostrongylus brasiliensis* and *Strongyloides ratti* (33) are mainly T-cell-mediated, accompanied by the production of interleukin (IL)-4, IL-5, IL-9, and IL-13 (34–36). These worm infections also promote goblet cell proliferation and increase the production and secretion of mucin (**Figure 2B**). The core structural components of mucus are MUC2 and FCGBP, and the other products of goblet cells include CLCA1 and ZG16. Therefore, the secretion of cytokines can promote the secretion of FCGBP. In dysentery caused by *Entamoeba histolytica* infection, FCGBP and MUC2 production increases significantly, preserving the integrity of the intestinal mucus layer and maintaining mucus gel protection (37). IL-22, a member of the cytokine IL-10 family, also has a similar effect (38). A significantly increased expression of FCGBP, CLCA1, and TFF3 was detected in the serum of patients suffering from ulcerative colitis and Crohn’s disease (39). Chronic stimulation by inflammatory cytokines and other destructive molecules damages the intestinal barrier function. Under these conditions, FCGBP acts as an antigen. It is recognized by macrophages and presented to T cells, activating the primary defense mechanisms (40).

Similar research reports that FCGBP inhibits the complement-mediated hemolysis of sheep red blood cells. The inhibition of complement-mediated responses to IgG molecules on mucosal surfaces or in external secretions is beneficial. Under normal circumstances, external secretions only contain small amounts of IgG (40). FCGBP can prevent complement-mediated damage by binding any available IgG. FCGBP is thought to act as a viral trap for HIV–antibody complexes (7). FCGBP makes IgG-viral complexes trap in the mucosa through binding to the Fc portion of IgG-viral complexes in order to prevent viral particles from reaching the epithelial tissue. For another, FCGBP facilitates the transfer of these complexes across the mucosa, where they are identified and eliminated by macrophages (**Figure 2C**). However, it still needs to be further explored whether T cells and Dendritic cells (DC) cells can also internalize the IgG-viral-FCGBP complex. Vaccine-induced polyclonal antibodies that recognize multiple viral epitopes can also promote the elimination of SARS-CoV-2 with the help of FCGBP (15). In addition to its role in innate immune defenses at the mucosal epithelium, FCGBP may also play a structural role. FCGBP has been reported to be expressed in the aorta, and it is the most downregulated gene in patients with ruptured abdominal aortic aneurysms (41). Genetic variations of FCGBP are also found in cerebral arteriovenous malformations (42). In conclusion, as part of the innate immune defense at the mucosal epithelium, FCGBP can regulate the attachment of pathogenic microorganisms to the mucosal surface, influencing disease progression (3).

Another epithelial, the pulmonary airway, is composed of the pseudostratified ciliated columnar epithelium. Here, mucus secreted by goblet cells and lung mucus gland cells provide lubrication, protecting lung tissue. While a reduction of this mucus barrier makes the lungs vulnerable to damage, mucus overproduction, or its reduced clearance, will lead to the onset of common airway diseases, such as cystic fibrosis, pulmonary fibrosis, asthma, and chronic obstructive pulmonary disease. PM10 is a major air pollutant promoting and aggravating allergic reactions and inflammation in the respiratory tract. Studies have found that the expression of FCGBP and MUC5AC increases significantly, by approximately 58-fold, when the animals of an ovalbumin- induced asthma mouse model are exposed to PM10 (43). FCGBP produced by bronchial mucus gland cells and goblet cells in children may be involved in the unique Ab response, recurrent infection, and the effects of serum therapy and vaccination (15). This may provide a potential explanation for the low infectivity and reduced lethality of SARS-CoV-2 in children (15). As both MUC5AC and FCGBP contain von Willebrand factor domains, it is speculated that FCGBP may play a similar role in acute lung injury as MUC5AC. In addition, the alternative splicing of FCGBP mRNA has been detected in patients with smoking-induced lung cancer (44), raising the possibility that this phenomenon may play a role in the pathogenesis of lung cancer. *Mycoplasma* infections can cause pneumonia, resulting in airway remodeling, bronchitis in the small airways, and occlusion bronchitis. This leads to bronchial epithelial cell proliferation, pronounced mucus secretion, and increased epithelial height. Thus, the degree of inflammation appears to be closely linked to the number and distribution of goblet cells. Moreover, the expression of FCGBP antigen in the serum of pneumonia patients decreased with the progression of the disease (45). Although the exact mechanism of FCGBP in the pathogenesis of *Mycoplasma pneumoniae* pneumonia is unknown, the role of this molecule in inflammation and progression cannot be ignored. Further studies on the anti-inflammatory action of FCGBP in bronchial epithelia will be necessary to resolve this issue.

### Interaction between IgGFc-binding protein and mucin-2

MUC2 is the main component of intestinal mucus. Together with FCGBP, MUC2 forms the intestinal mucus reticular scaffold structure, forming an important part of the first line of defense during innate immune responses. In 2009, Johansson and his team reported that the N-terminal end of FCGBP was covalently linked to MUC2, contributing to the crosslinking and stability of the mucin network (22). However, in 2021, while conducting a sensitive mass spectrometry of mouse mucus isolated under non-reducing conditions, Erik et al. could not confirm that FCGBP was covalently bound to other mucus proteins, such as MUC2 (16). Moreover, as a mucin secreted by goblet cells, the majority of FCGBP was present in the soluble component of the intestinal mucus layer, while MUC2 mainly appeared as an insoluble component (16). If assumptions made earlier had been correct, the heterodimers of FCGBP and MUC2 should have been detected in abundance. Such contradictory observations could be caused by many factors. Firstly, FCGBP may be covalently bound to other mucus proteins other than MUC2, or post-translational modification of MUC2, such as its ST6GALNAC1 (ST6)-mediated sialylation could affect interactions with FCGBP (46). Secondly, it is unclear how the reactive C-terminus formed during the cleavage of the GDPH sequence could covalently bind to MUC2. Thirdly, it is possible that FCGBP interacts with MUC2 only briefly rather than becoming crosslinked to the highly insoluble MUC2 mucin framework. FCGBP could maintain the integrity of the structure of MUC2 by stabilizing the mucus layer. This integrated mucus layer can respond to infection by *E. histolytica* in colonic pathogenesis. Dynamic interactions between mucin and its interacting proteins, including FCGBP, can help to maintain its role in innate immune responses at the mucosal surface of the intestine and airways (22).

### Interactions of IgGFc-binding protein and trefoil factor families

Peptides in the mammalian trefoil factor family (TFF) include TFF1, TFF2, and TFF3. The three molecules have a similar structure containing conserved cysteine residues and intramolecular disulfide bonds (CysI-V, CysII-IV, and CysIII-V) (47, 48). TFF1 and TFF3 are distributed differently in the body. TFF1 is synthesized in the stomach, while TFF3 is mainly found in the intestine and in salivary glands (49). FCGBP secreted by intestinal goblet cells and salivary gland mucus acini combine with TFF1 and TFF3, respectively, to form heterodimers linked by disulfide bonds (6). Overall, the TFF3-FCGBP dimer is being produced more abundantly (50). Colonic mucus is a double-layered structure that consists of an insoluble inner layer and a soluble outer layer. Under physiologic conditions, bacteria do not pass through the insoluble inner layer. However, in the dextran sodium sulfate (DSS)–induced mouse colitis model, the reduced thickness of the mucus allows bacteria to penetrate this inner layer, reaching the epithelium and causing inflammation (51). The complexes of TFF1-FCGBP in gastric mucus and TFF3-FCGBP in colonic mucus are present in the soluble layer, and their molecular function appears similar (52). The TFF3-FCGBP heterodimer is part of the innate immune defense of the mucosal epithelium in the intestine and airways, preventing microbial infiltration and trapping viruses with the help of immunoglobulins on mucosal surfaces (50). The cysteine-rich structure supports the function of FCGBP in eliminating microorganisms, and even the N-terminal domain of some bacterial proteins is homologous to the N-terminal domain of FCGBP (53) (**Figure 2D**). As TFF3 is a lectin capable of binding to microorganisms, its heterodimerization with FCGBP results in synergistically enhancing or modulating the binding of microorganisms (54). For example, lipopolysaccharide, which recognizes *Helicobacter pylori*, shows a potential antibacterial activity (54). The presence of TFF3-FCGBP heterodimers is not only limited to the gastrointestinal tract, but also found in human saliva, lung, and cervix (55). Thus, heterodimers formed by FCGBP and TFF family members strengthen the barrier function of the outer layer of colonic mucus. These complexes inhibit bacterial attachment and movement and support their clearance, preventing bacteria from penetrating into the inner layer of mucus (50). TFF3-FCGBP heterodimers play a unique role in innate immunity by strictly controlling the adhesion of pathogens to the mucosal surface.

## Relevance of IgGFc-binding protein to cancer

### Promoting tumorigenesis and metastasis

The overexpression and altered glycosylation of tumor-associated mucins mediate the interaction between cancer cells and leukocytes in the tumor microenvironment. This results in the activation of innate immune effector function, stimulates inflammation, and promotes the scattering of tumor cells, leading to distant colonization (56). Abnormal glycosylation leads to the changes in the structure of tumor-associated mucins, including MUC1 and MUC2, contributing to metastasis formation. The deletion of MUC2 stimulates tumor-associated macrophages to produce more IL-6, which leads to the increase of STAT3 signal transduction and epithelial mesenchymal transformation (EMT) by colon cancer cells (57). There are many proteins capable of inducing EMT in tumor cells, including transforming growth factor-β (TGF-β). FCGBP is the most profoundly downregulated transcript during TGF-β-induced EMT in gallbladder cancer cells, suggesting that it may play an important role in this process (14). At the same time, patients with tumors expressing high levels of FCGBP are more likely to suffer from invasive growth and metastatic recurrence. Univariate Kaplan–Meier survival analysis shows that the expression of FCGBP correlates significantly with the mean survival time in patients with gallbladder cancer. Multivariate Cox analysis shows that the reduced FCGBP expression impacts negatively on survival. Therefore, the expression of FCGBP can be used as a tool for the early detection and population screening of gallbladder lesions (14). FCGBP expression is an independent indicator of disease progression, clinical biological behavior, and prognosis in patients with gallbladder cancer. Thus, the modulation of FCGBN transcription may become a potential therapeutic target (14).

Metastasis formation remains an important factor in most cancer-related deaths as the presence of metastatic lesions limits the effectiveness of available therapeutic options. Cell adhesion in the vasculature of specific organs is a critical step in tumor metastasis, and FCGBP expression significantly increases cell adhesion (58). This suggests that FCGBP may play an important role in colorectal cancer (CRC) metastasis by participating in cell adhesion (59). Osteosarcoma is the most common primary malignant bone tumor in children and adolescents. It attracts attention because of its highly invasive behavior and its tendency to form early systemic metastases. Using microarray technology, bioinformatics, and weighted gene coexpression analysis, FCGBP has been identified as a core gene in the metastasis formation of osteosarcomas (60).

Some researchers have showed that the protein and mRNA expression levels of FCGBP decrease with the progression of the disease in the primary tissues and liver metastases of colon cancer. The expression of a key mucin, MUC2, shows a similar decreasing pattern. The concomitant downregulation of these two molecules leads to the destruction of the epithelial barrier and promotes the invasion and metastasis of colon cancer (61, 62). Because FCGBP and MUC2 have highly similar domains, it is tempting to speculate that FCGBP plays a role in promoting the invasion and metastasis of colon cancer.

### IgGFc-binding protein as a biomarker for cancer treatment and prognosis

The term “autoimmune disease” refers to conditions caused by an immune response against self-antigens. Although these antigens are present in the body, they do not induce an immune response under normal circumstances. However, the immune system starts to produce autoantibodies and cellular responses, which will eventually induce tissue damage if this immune tolerance collapses. A wide variety of autoimmune diseases, including ulcerative colitis and Crohn’s disease in the gastrointestinal tract, have been recognized. In these conditions, the chronic inflammatory response stimulates effector cells to produce chemotactic cytokines and inflammatory cytokines. The resulting damage to the epithelial barrier also affects intestinal epithelial cells and goblet cells (63, 64). Once this barrier is compromised, the immune system starts to produce autoantibodies. FCGBP secreted by goblet cells, as an antigenic substance, binds to various subtypes of IgG Fc and secretes into the gastrointestinal tract together with other mucosal proteins (65). The level of FCGBP antigen in the serum of patients with ulcerative colitis and Crohn’s disease reflects the pathophysiologic state and can be used as a diagnostic marker for these autoimmune diseases (40).

The differential expression of FCGBP in various tumors may indirectly provide clues to its potential function and significance. Recent studies have found that FCGBP is expressed in 33 carcinomas compared with surrounding normal tissues (66). Moreover, it is significantly highly expressed in invasive breast carcinoma, diffuse large B-cell lymphoma, glioblastoma multiforme, lung adenocarcinoma, pancreatic adenocarcinoma, rectal adenocarcinoma, ovarian carcinoma, lower grade glioma, gastric cancer, and Human Papillomavirus (HPV)-infected patients with head and neck squamous cell carcinoma (HNSCC) (66–69). Of these, the overexpression of this molecule usually predicts poor prognosis in ovarian carcinoma and lower-grade glioma (67). However, higher expression is associated with longer survival time in HPV-infected patients with HNSCC (70). FCGBP is detected in the amniotic fluid of pregnant women suffering from preterm labor with ruptured membranes and intact membranes. In addition, FCGBP is also detected in amniotic fluid during the microbial invasion of the amniotic cavity and acute histologic chorioamnionitis. Thus, Stranik speculates that FCGBP may be used as a novel diagnostic marker for intraamniotic infection in the two forms of natural preterm births (10). In contrast, a significant downregulation of FCGBP is found in adrenocortical carcinoma, chromophobe kidney cancer, clear cell kidney carcinoma, HNSCC and rectum adenocarcinoma, and cutaneous skin melanoma (66–68). Similarly, the expression of FCGBP is downregulated in colon cancer than that of surrounding normal tissues. Furthermore, the expression of FCGBP is even lower in metastatic lesions compared with the primary tumors. Thus, FCGBP may be used as a prognostic marker for survival in metastatic colon cancer. Rajkumar et al. show that FCGBP is highly expressed in gastric cancer and it is involved in the development of these tumors (69). FCGBP also shows reduced expression in thyroid adenomas and thyroid cancers compared with normal thyroid tissue. Thus, FCGBP expression levels may help to distinguish between thyroid follicular adenomas and follicular carcinomas (71, 72). FCGBP expression is reduced in human prostate cancers and the transgenic adenocarcinoma of mouse prostate cancer (TRMAP) disease model (73). A reduced expression of FCGBP may reflect its potential role in prostate malignancy and may be an indicator for progression (74). As Li et al. reported, decreased FCGBP in gallbladder carcinoma cell line (GBC-SD cells) suggests that FCGBP is an important marker for clinical biological behavior and progression (14). The above results all indicate that FCGBP may have the potential to be used as a diagnostic biomarker and prognostic factor for cancers (**Table 1**).

**Table 1:** IgGFc-binding protein (FCGBP) as a prognostic or diagnosis marker for disease.

Type of disease	Expression state	Function
Autoimmune diseases	Upregulation	Markers of diagnostic (40)
Ovarian carcinoma	Upregulation	Markers of poor prognosis (67)
Lower-grade glioma	Upregulation	Markers of poor prognosis (67)
HPV-infected HNSCC	Upregulation	Markers of good prognosis (70)
Gastric cancer	Upregulation	Participate in the occurrence and development of gastric cancer (69)
Premature intraamniotic infection	Upregulation	Biodiagnostic markers (10)
HNSCC	Downregulation	Markers of poor prognosis (68)
Rectum adenocarcinoma	Downregulation	Markers of poor prognosis (70)
Colon adenocarcinoma	Downregulation	Markers of poor prognosis (75)
Metastatic colon cancer	Downregulation	Markers of poor prognosis (76)
Thyroid cancer	Downregulation	Distinguish between follicular adenomas and follicular carcinomas (71)
Prostate cancer	Downregulation	Markers of poor prognosis (74)
Gall bladder cancer	Downregulation	Markers of poor prognosis, tools for early detection of gallbladder cancer in benign lesions (14)

### Effects on tumor immune responses

Immune cells in the tumor microenvironment are key components of tumor tissue, and accumulating evidence supports the clinicopathologic significance of immune cell infiltration in predicting the survival and treatment responsiveness of tumor patients (77, 78). As the expression patterns of FCGBP shows a marked variability in different tumors, its role in carcinogenesis may differ significantly. Kai et al. have conducted a functional clustering and pathway analysis of the molecules influenced by FCGBP. The GO results show that FCGBP is involved in immune-related pathways, such as leukocyte-mediated immune regulation and B- and T-cell activation. Additional KEGG pathway analysis shows that FCGBP is involved in chemotaxis, RAP1 signaling, T-cell leukemia virus infection, and B-cell receptor signaling (67). This analysis indicates the involvement of FCGBP in a wide range of immune- and inflammation-related biological processes.

It is reported that FCGBP protein abundance correlates with M2 macrophage responses, B-cell, macrophage, and DC infiltration. For example, in ovarian cancer, FCGBP protein expression correlates with the expression of the macrophage marker CD163, Mrc1, and TGFβ1. In the other work, FCGBP correlates positively with M2 macrophage polarization and negatively with M1 polarization (67). This suggests that FCGBP is also involved in regulating the infiltration of immune cells in the tumor microenvironment of ovarian cancer, where it may be a valuable marker for prognostic evaluation (67, 75). In CRC, FCGBP abundance shows inverse correlation with tumor purity and macrophage expression but coincides with a high number of B cells, suggesting that FCGBP may be involved in humoral immune responses in this cancer (76). After adjusting for tumor purity, 51 of 57 immune cell markers shows significant positive correlation with FCGBP, suggesting that FCGBP plays an important role in immune cell infiltration into the microenvironment of CRC. High FCGBP protein abundance contributes to increased recruitment of immune cells in low-grade gliomas in these nervous system malignancies (66). Immune checkpoint inhibitors and tumor vaccines represent promising approaches in tumor immunotherapy. An mRNA vaccine designed to target FCGBP has shown some promise in the treatment of low-grade gliomas (79). At the same time, the positive correlation between FCGBP protein abundance and PD-L1 and CD8 expression also suggests that FCGBP may be involved in the upregulation of immune checkpoints and has the potential to become a new immunotherapy biomarker (66).

## Conclusions

Although the existence of FCGBP has been reported for more than 20 years, and a key role being suggested in regulating the tumor immune microenvironment, the physiologic functions of FCGBP are still not fully elucidated in intestinal mucosal defense and in cancer biology. For example, two particularly interesting phenomena need to be further explored. First, the elevated expression of FCGBP may reflect pathophysiologic changes in Crohn’s disease and ulcerative colitis (40), but the mRNA and protein expression of FCGBP are decreased in colorectal adenomas and cancers. Furthermore, cancer progression and the formation of metastatic lesions leads to further significant reduction (75), highlighting the role of FCGBP in oncogenesis and cancer progression. The second one is that the alternative splicing of FCGBP mRNA is detected in lung cancer (44), hepato-cholangiocarcinoma (80), CRC (75, 81), and brain arteriovenous malformations (42). These results raise the possibility that the heterozygous mutations of FCGBP may be potentially pathogenic. Clarifying these questions will help us understand the relevance of FCGBP in inflammatory disorders and cancer. It is also a significant research direction to establish whether FCGBP may be used as a marker for the early diagnosis and prognosis of disease processes.

## Author contributions

QL and LG contributed the research concept and design. QL drafted the manuscript. LG and YL revised and corrected the manuscript. XN, JZ, and SZ drafted the figures. QY and WZ re-edited the figures. All authors contributed to the article and approved the submitted version.

## Conflict of interest

The authors declare that the research was conducted in the absence of any commercial or financial relationships that could be construed as a potential conflict of interest.

## Publisher’s note

All claims expressed in this article are solely those of the authors and do not necessarily represent those of their affiliated organizations, or those of the publisher, the editors and the reviewers. Any product that may be evaluated in this article, or claim that may be made by its manufacturer, is not guaranteed or endorsed by the publisher.
